# Supplementary figures and images for: Multi-scale modeling of macrophage—T cell interactions within the tumor microenvironment
Source: PLoS Comput Biol. 2020 Dec 23;16(12):e1008519. doi: 10.1371/journal.pcbi.1008519 (PMC7790427; doi:10.1371/journal.pcbi.1008519)

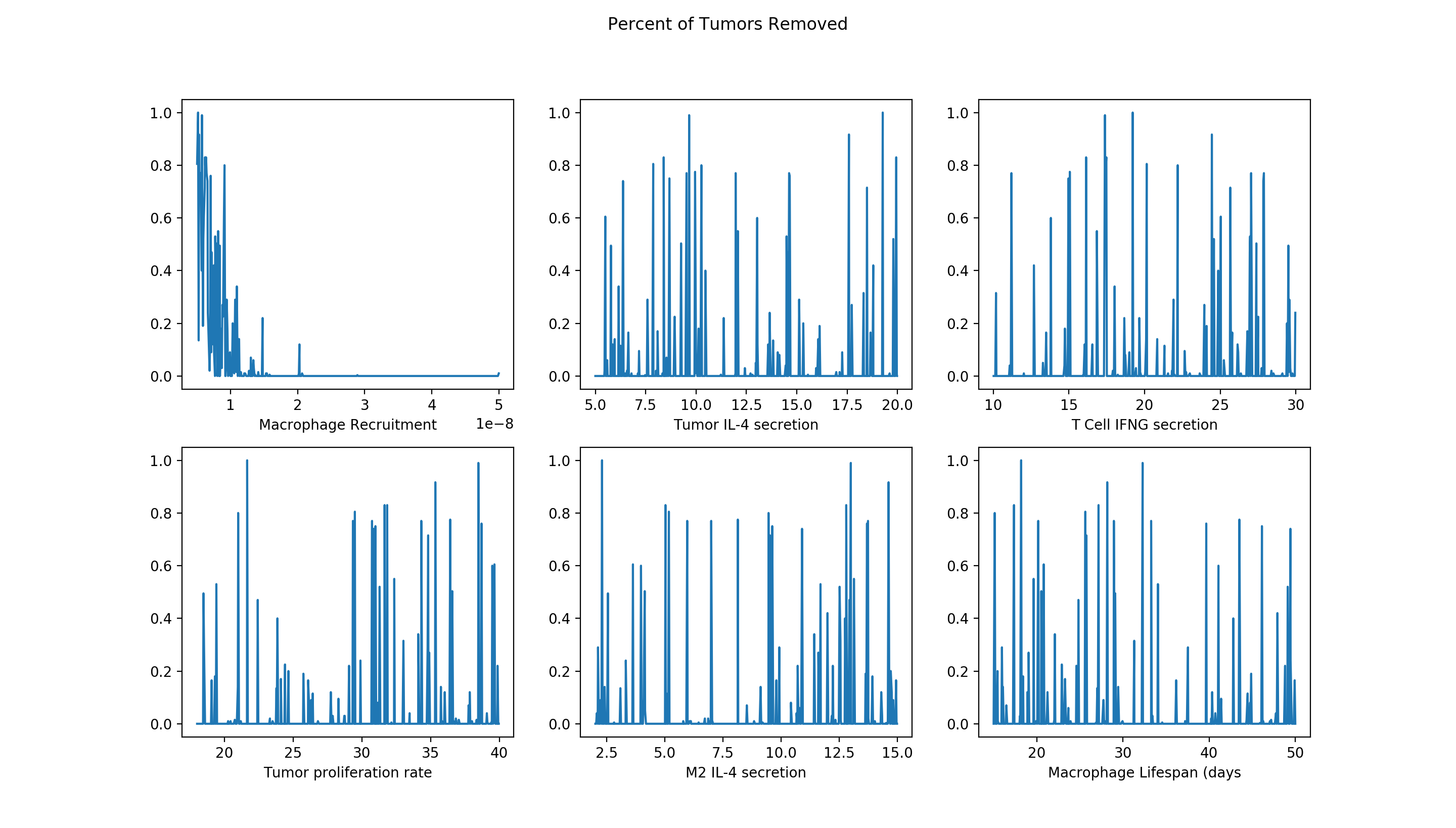

Supplement: S1 Fig — The fraction of tumors removed as a fraction of relevant tumor microenvironment parameters: macrophage recruitment rate, tumor IL-4 secretion rate, T cell IFN-γ secretion rate, tumor proliferation rate, M2 IL-4 secretion rate, and macrophage lifespan. With Latin Hypercube Sampling, parameter sets are sampled so that each parameter value appears only once. This is done for the sake of computational efficiency. Because only one parameter set was simulated for each parameter value, the plots are very discontinuous and do not show the average model behavior for each parameter value. Despite this, a clear trend is visible for macrophage recruitment rate. (TIF) [file pcbi.1008519.s001.tif]

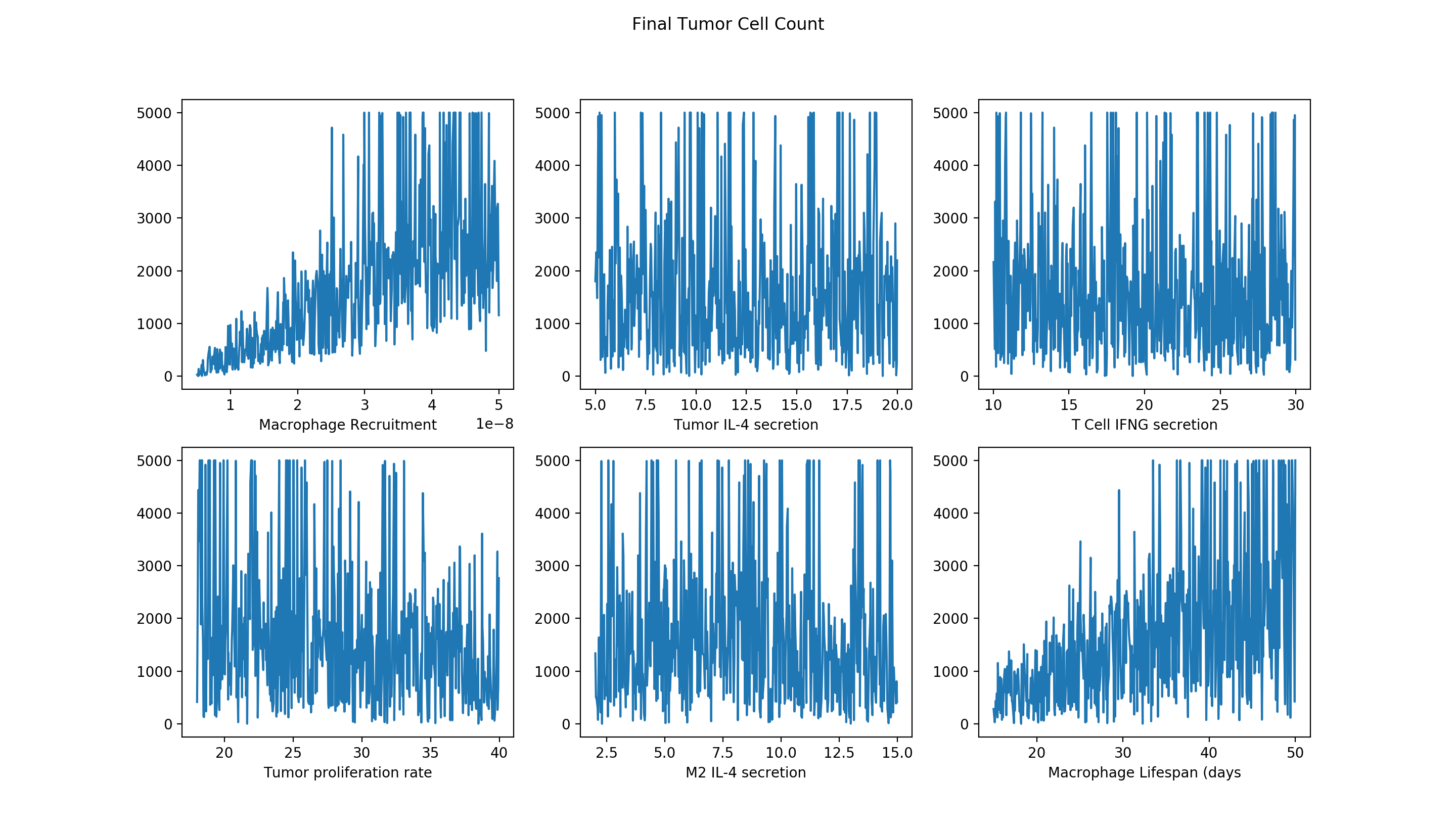

Supplement: S2 Fig — The final number of tumor cells is on the y-axis and the relevant parameters are on the x-axes (macrophage recruitment rate, tumor IL-4 secretion rate, T Cell IFN-γ secretion rate, tumor proliferation rate, M2 IL-4 secretion rate, and macrophage lifespan). Because only one parameter set was simulated for each parameter value, the plots are very discontinuous. However, a clear trend is visible for macrophage recruitment rate and macrophage lifespan. The maximum number of tumor cells here is 5,000 because we chose to end simulation when either maximum simulation time was reached, the tumor was eliminated, or tumor cell count reached 5,000. This is because at that number of tumor cells, there is much less space available for recruited immune cells, so their recruitment inherently decreases due to the nature of the model. (TIF) [file pcbi.1008519.s002.tif]

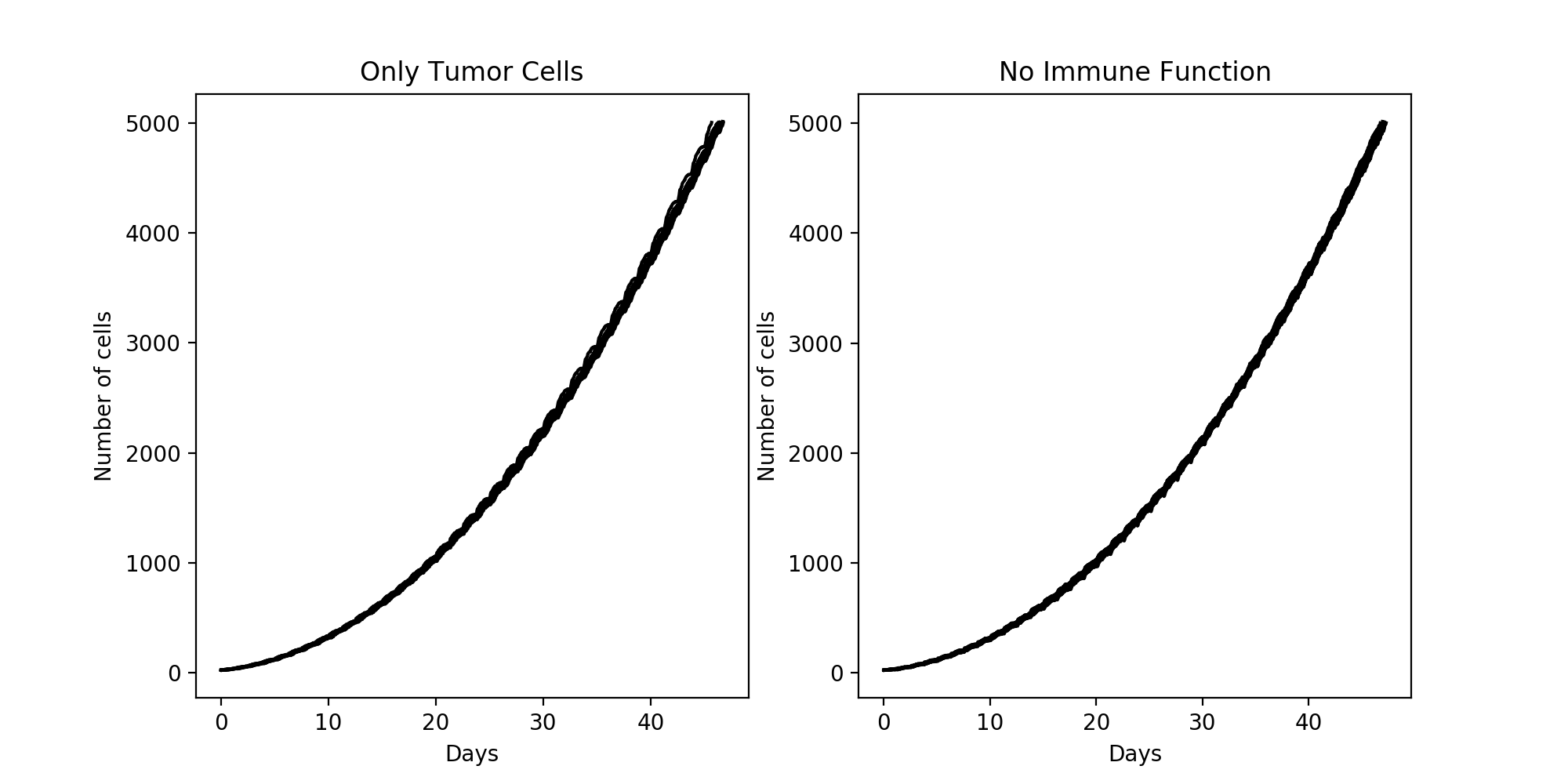

Supplement: S3 Fig — Comparison of tumor growth curves when there are no immune cells present in the simulation and when immune cells are present but lack function. Because these curves are very similar, we conclude that spatial inhibition is not the cause of the equilibrium state seen without treatment. Simulations were done in replicates of 10. (TIF) [file pcbi.1008519.s003.tif]

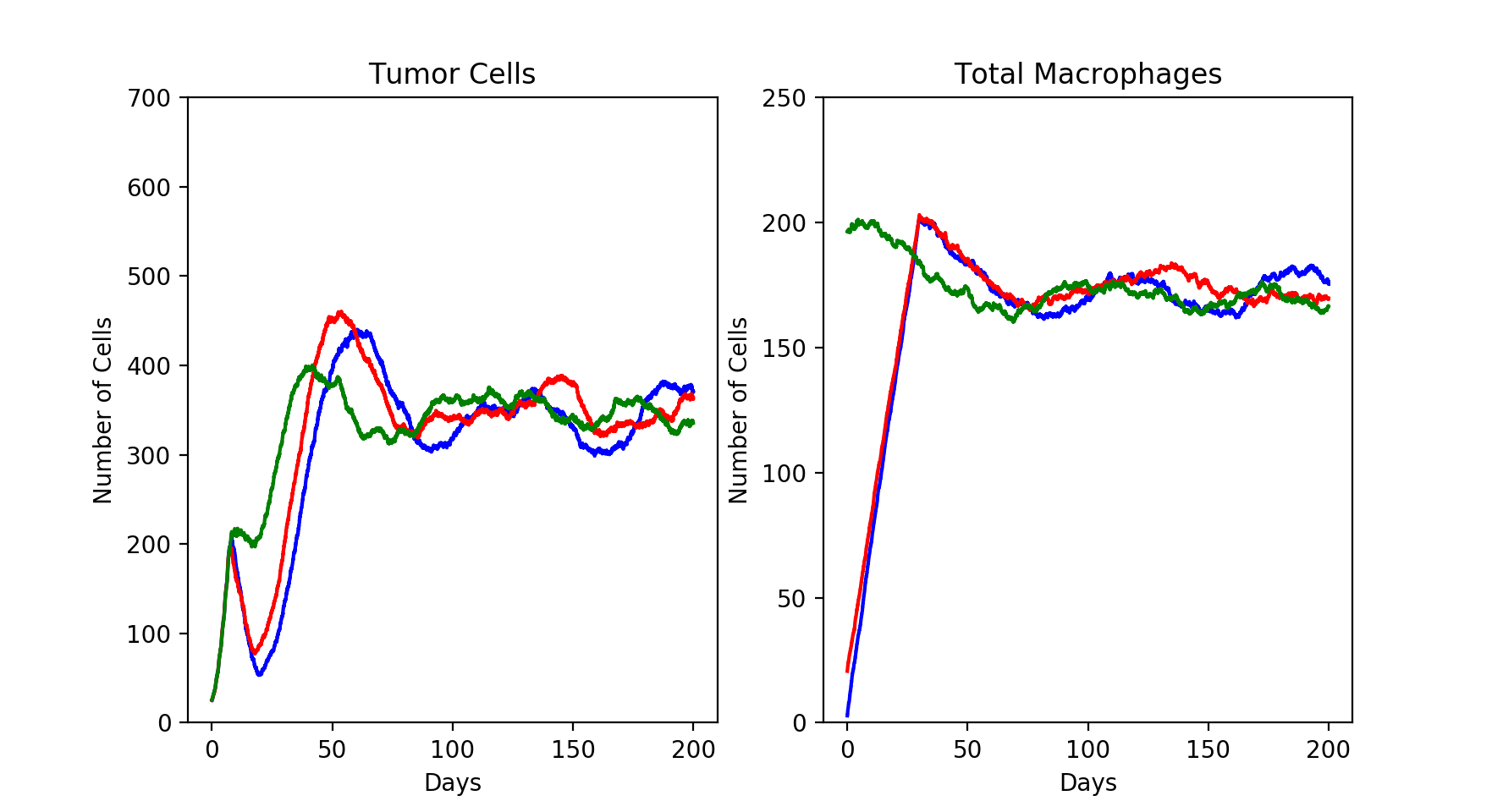

Supplement: S4 Fig — Comparison of tumor curves and total macrophage curves for differing starting macrophage densities: 2x10^-4^ (blue), 2x10^-3^ (red, value used for rest of the simulations), and 2x10^-2^ (green) cells per site. Each density was simulated in replicates of 10. Plotted are the average time courses for those replicates. (TIF) [file pcbi.1008519.s004.tif]

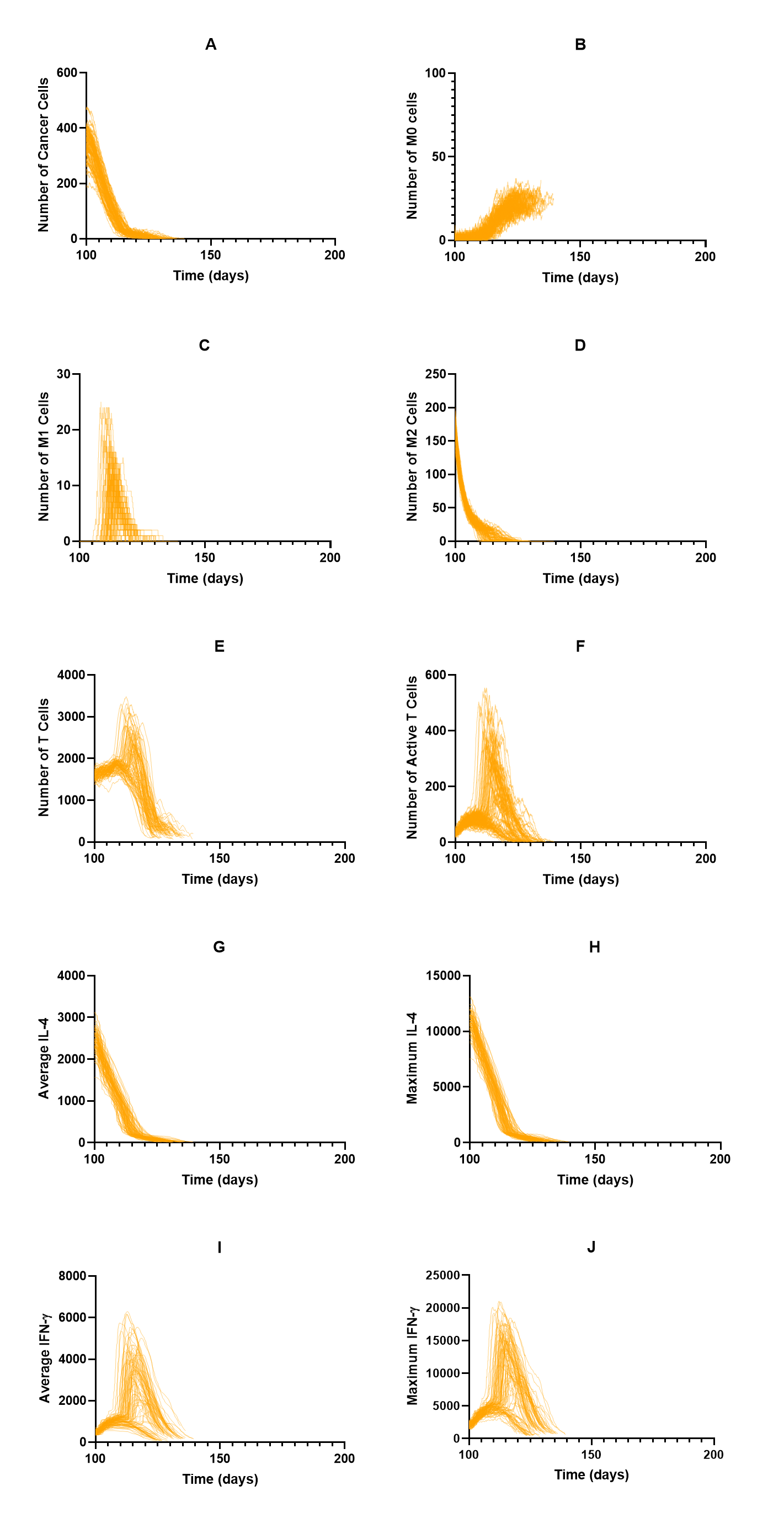

Supplement: S5 Fig — Tumors that were eliminated are shown in orange. (A) Cancer cells, (B) M0 macrophages, (C) M1 macrophages, (D) M2 macrophages, (E) T cells, (F) Active T cells, (G) Average IL-4, (H) maximum IL-4, (I) average IFN-γ, (J) Maximum IFN-γ. (TIF) [file pcbi.1008519.s005.tif]

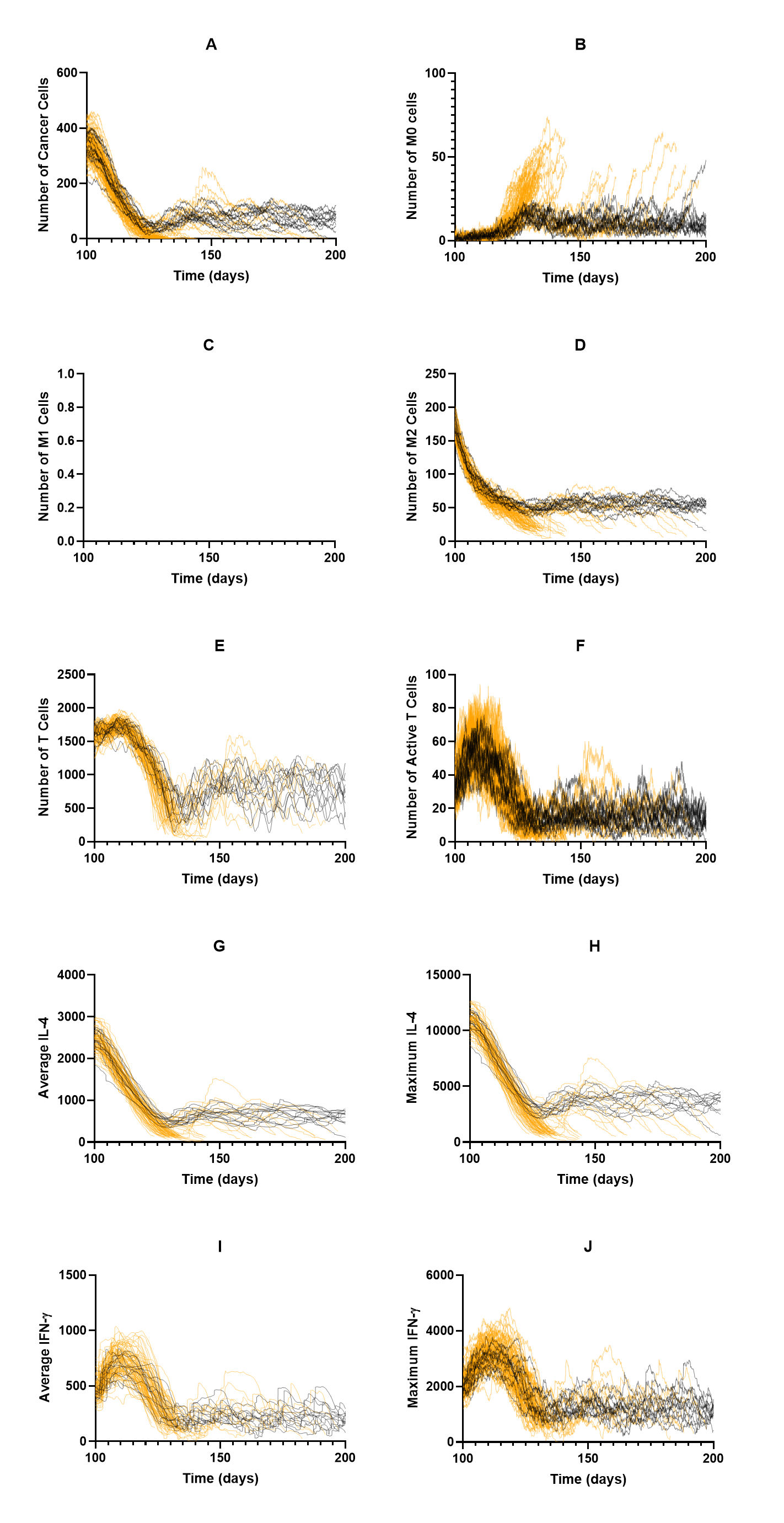

Supplement: S6 Fig — Tumors that survived to the end of simulation are shown in black. Tumors that were eliminated are shown in orange. (A) Cancer cells, (B) M0 macrophages, (C) M1 macrophages, (D) M2 macrophages, (E) T cells, (F) Active T cells, (G) Average IL-4, (H) maximum IL-4, (I) average IFN-γ, (J) Maximum IFN-γ. (TIF) [file pcbi.1008519.s006.tif]

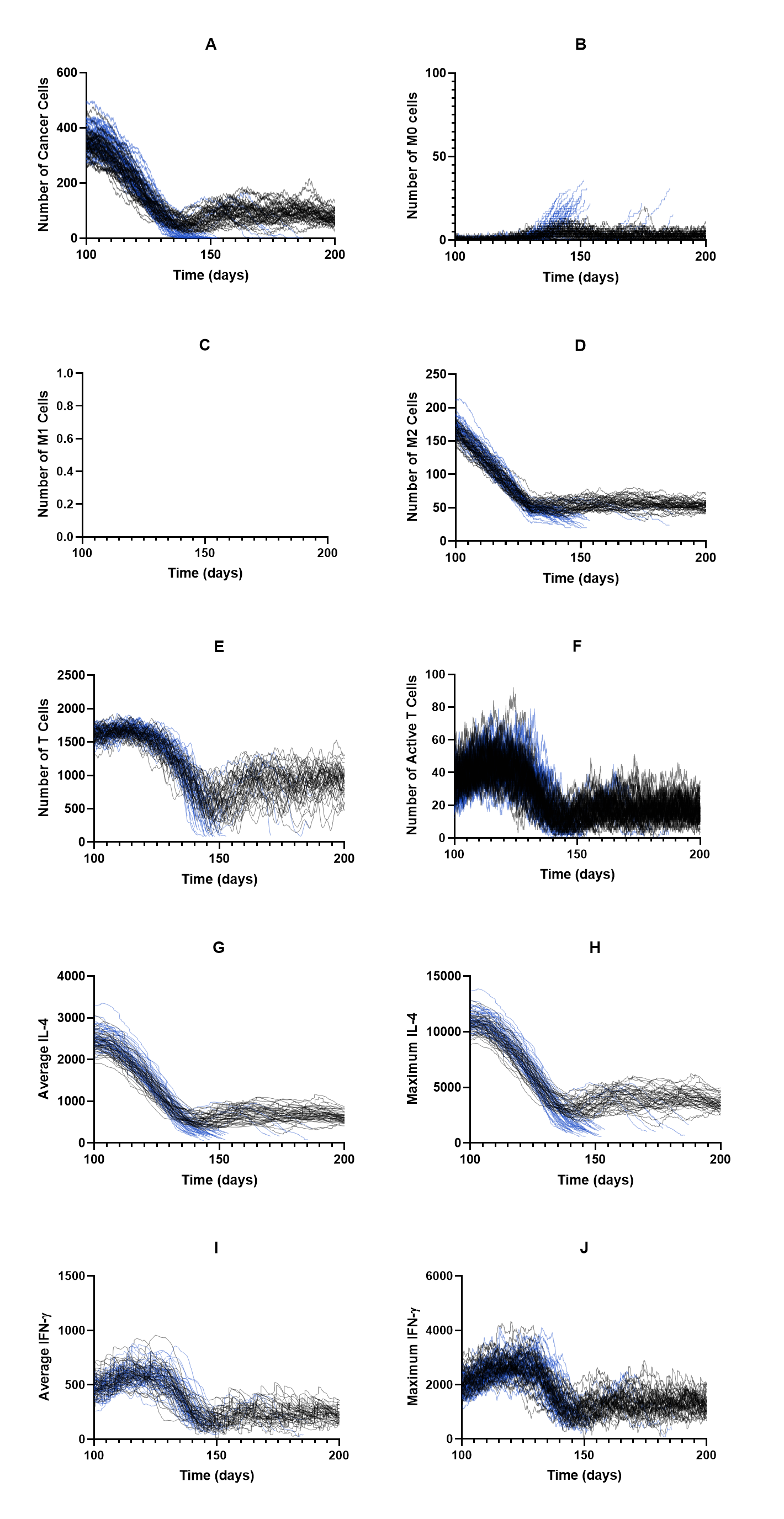

Supplement: S7 Fig — Tumors that survived to the end of simulation are shown in black. Tumors that were eliminated are shown in blue. (A) Cancer cells, (B) M0 macrophages, (C) M1 macrophages, (D) M2 macrophages, (E) T cells, (F) active T cells, (G) Average IL-4, (H) Maximum IL-4, (I) Average IFN-γ, (J) Maximum IFN-γ. (TIF) [file pcbi.1008519.s007.tif]

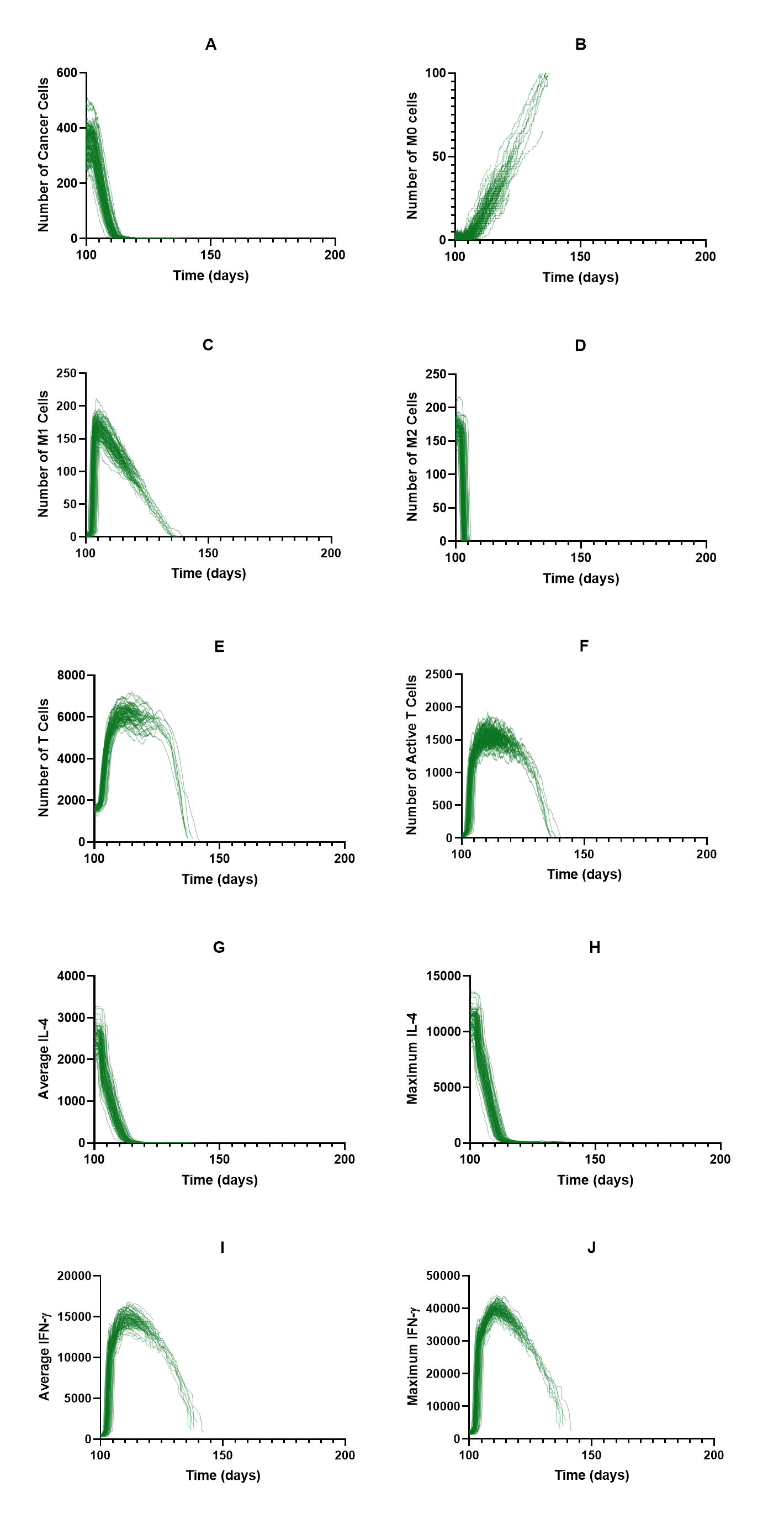

Supplement: S8 Fig — Tumors that survived to the end of simulation are shown in black. Tumors that were eliminated are shown in green. (A) cancer cells, (B) M0 macrophages, (C) M1 macrophages, (D) M2 macrophages, (E) T cells, (F) active T cells, (G) Average IL-4, (H) Maximum IL-4, (I) Average IFN-γ, (J) Maximum IFN-γ. (TIF) [file pcbi.1008519.s008.tif]

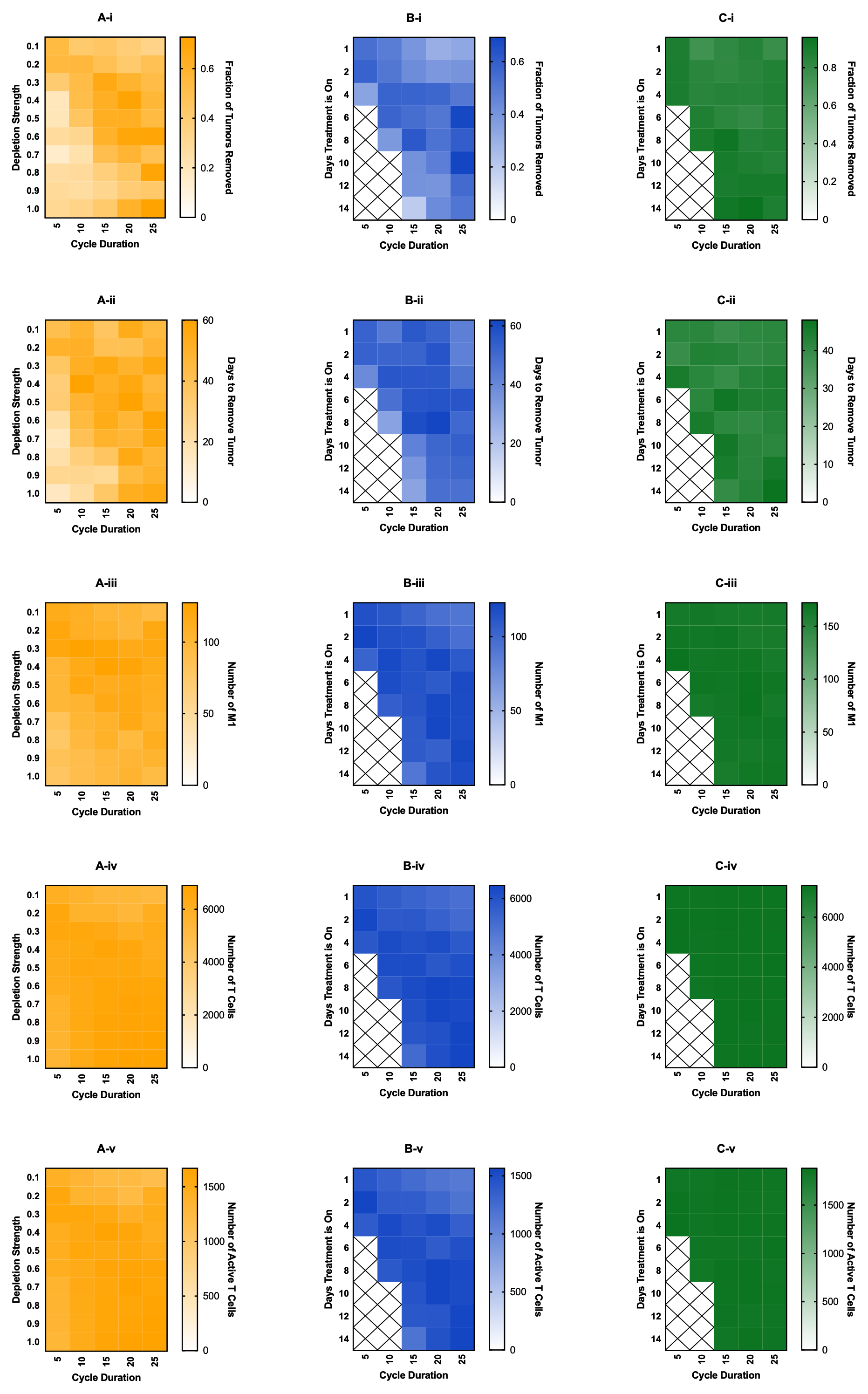

Supplement: S9 Fig — For macrophage depletion (A), the fraction of macrophages removed at the beginning of each cycle is given as “Depletion Strength” and the length of each cycle is “Cycle Duration.” For recruitment inhibition (B) and PI3K inhibition (C), the number of days in the cycle that treatment is on for is given as “Days Treatment is On.” Recruitment inhibition is simulated at a strength of 1.0 (complete inhibition) and PI3K inhibition is simulated at a strength of 0.8. For recruitment inhibition and PI3K inhibition, spaces marked with an X are those where treatment-on time is equal or greater to the cycle duration, thus were not simulated. (i) fraction of tumors removed after starting therapy. (ii) time (days) from starting treatment to tumor removal. It is averaged over the 100 simulations and is equal to zero if no tumors were removed at that treatment level. (iii) maximum number of M1 macrophages. (iv) maximum number of total T cells. (v) maximum number of active T cells. (TIF) [file pcbi.1008519.s009.tif]

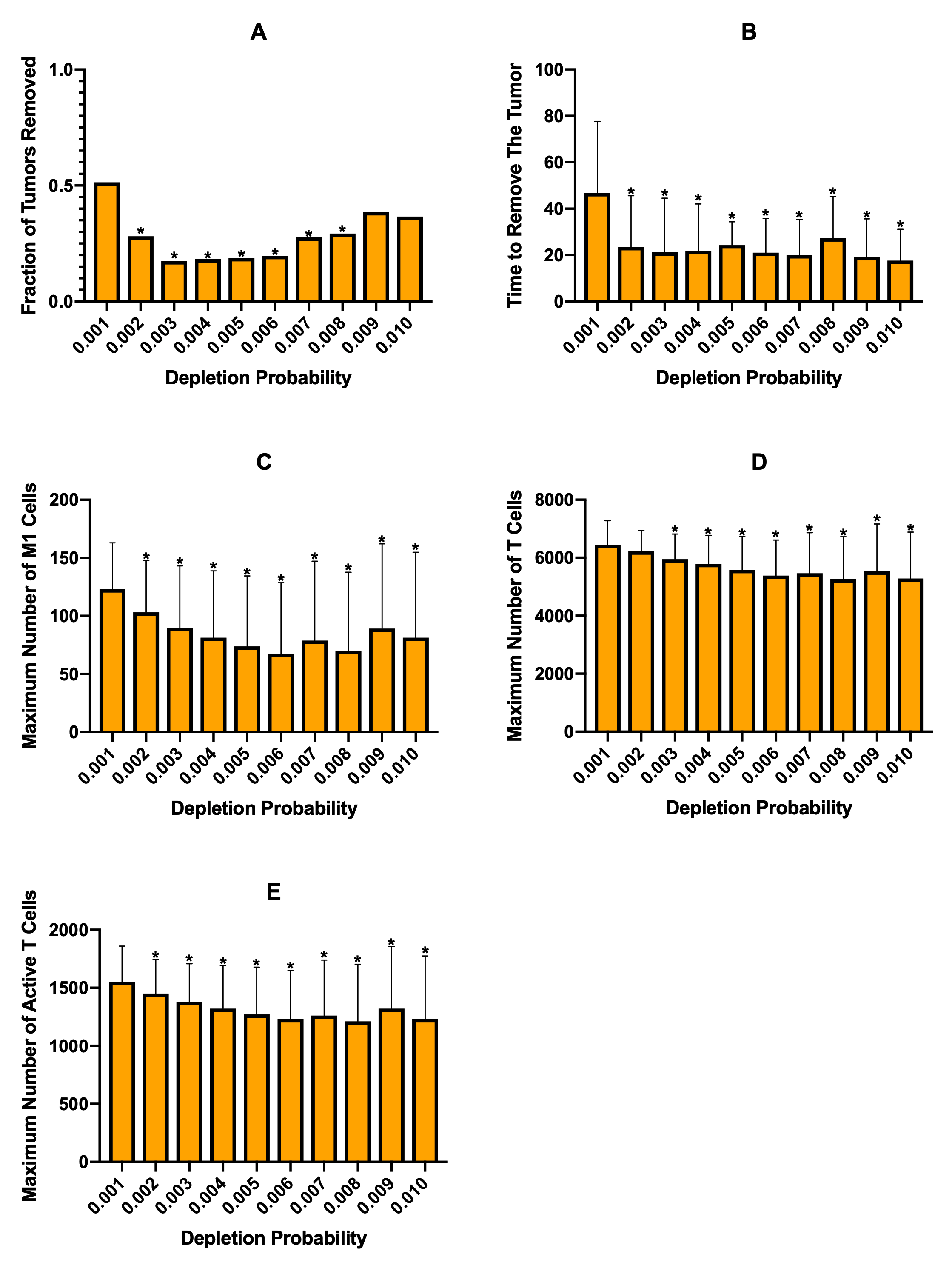

Supplement: S10 Fig — (A) fraction of tumors removed after starting simulation, (B) average time needed to remove the tumor, (C) the maximum number of M1 macrophages, (D) the maximum number of total T cells, (E) the maximum number of active T cells. Note the differences in y-axis scales across treatment strategies. Asterisks signify that a result is statistically significant (p<0.01) from the result of the lowest treatment strength. We note that for the time needed to remove the tumor (B), plotted is the time averaged over only simulations where the tumor was removed. Therefore, while some bars may appear much higher than that of the lowest treatment strength, they only represent a small number of simulations out of 100 and thus were not found to be statistically significant. (TIF) [file pcbi.1008519.s010.tif]

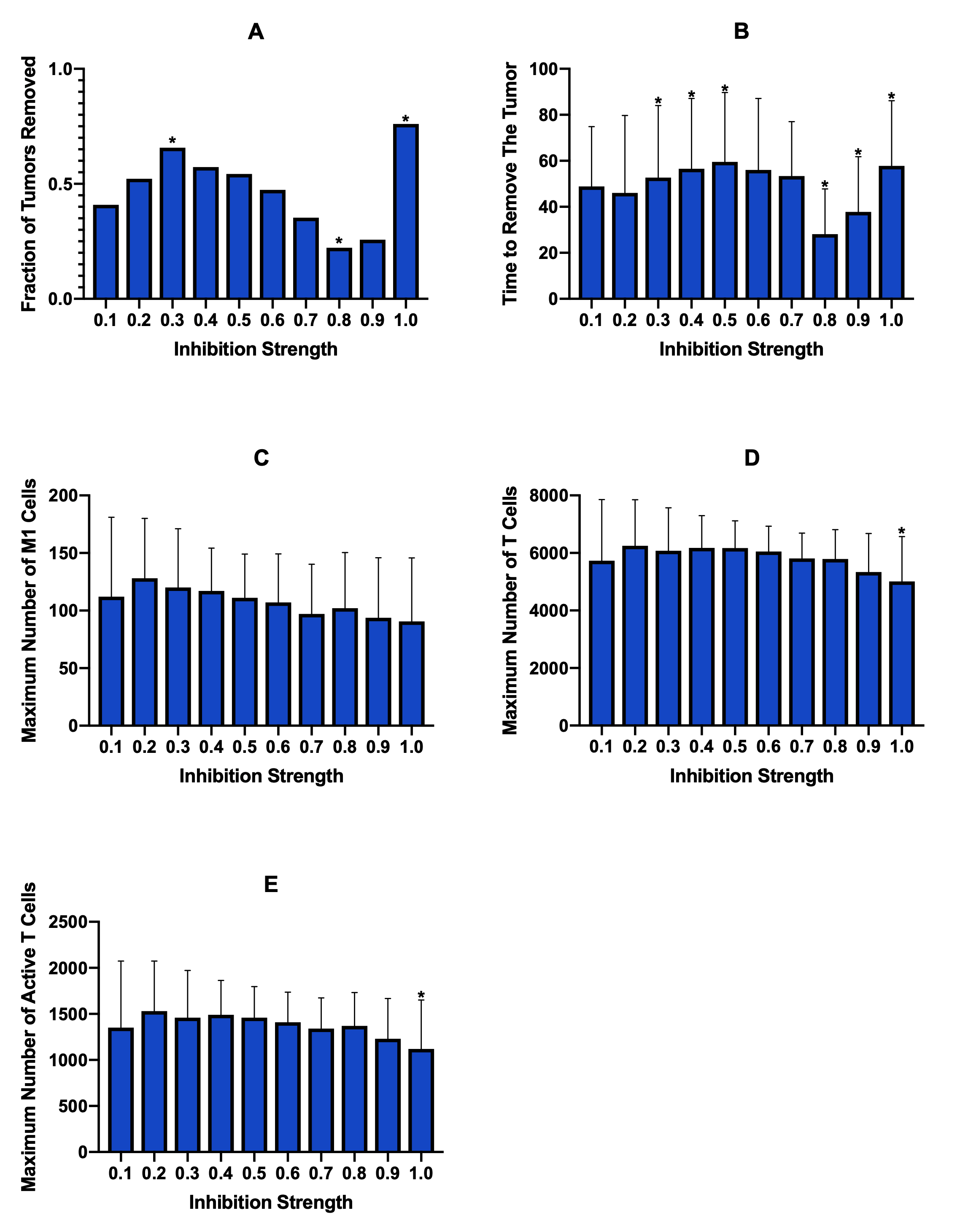

Supplement: S11 Fig — (A) fraction of tumors removed after starting simulation, (B) average time needed to remove the tumor, (C) the maximum number of M1 macrophages, (D) the maximum number of total T cells, (E) the maximum number of active T cells. Note the differences in y-axis scales across treatment strategies. Asterisks signify that a result is statistically significant (p<0.01) from the result of the lowest treatment strength. We note that for the time needed to remove the tumor (B), plotted is the time averaged over only simulations where the tumor was removed. Therefore, while some bars may appear much higher than that of the lowest treatment strength, they only represent a small number of simulations out of 100 and thus were not found to be statistically significant. (TIF) [file pcbi.1008519.s011.tif]

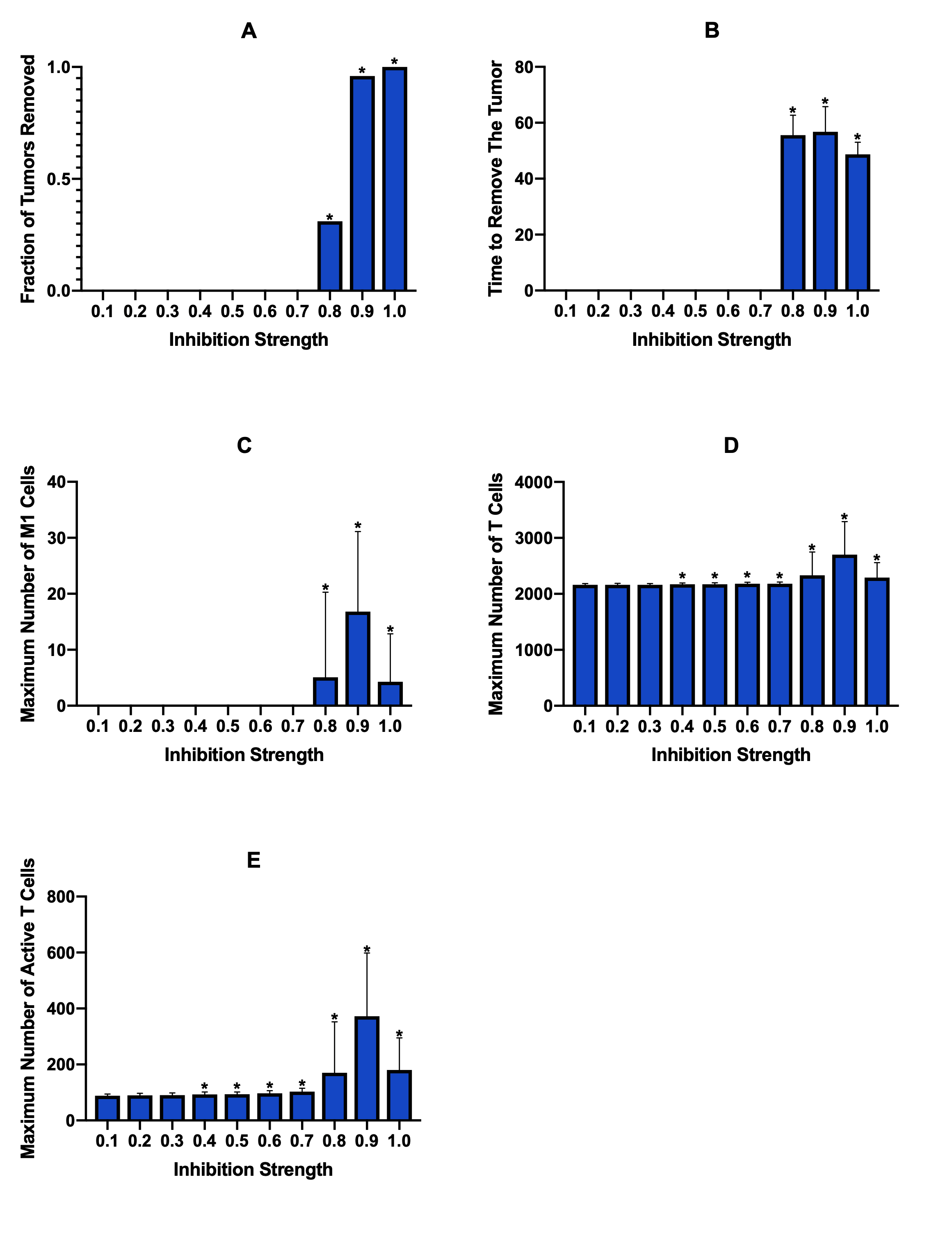

Supplement: S12 Fig — (A) fraction of tumors removed after starting simulation, (B) average time needed to remove the tumor, (C) the maximum number of M1 macrophages, (D) the maximum number of total T cells, (E) the maximum number of active T cells. Note the differences in y-axis scales across treatment strategies. Asterisks signify that a result is statistically significant (p<0.01) from the result of the lowest treatment strength. We note that for the time needed to remove the tumor (B), plotted is the time averaged over only simulations where the tumor was removed. Therefore, while some bars may appear much higher than that of the lowest treatment strength, they only represent a small number of simulations out of 100 and thus were not found to be statistically significant. (TIF) [file pcbi.1008519.s012.tif]

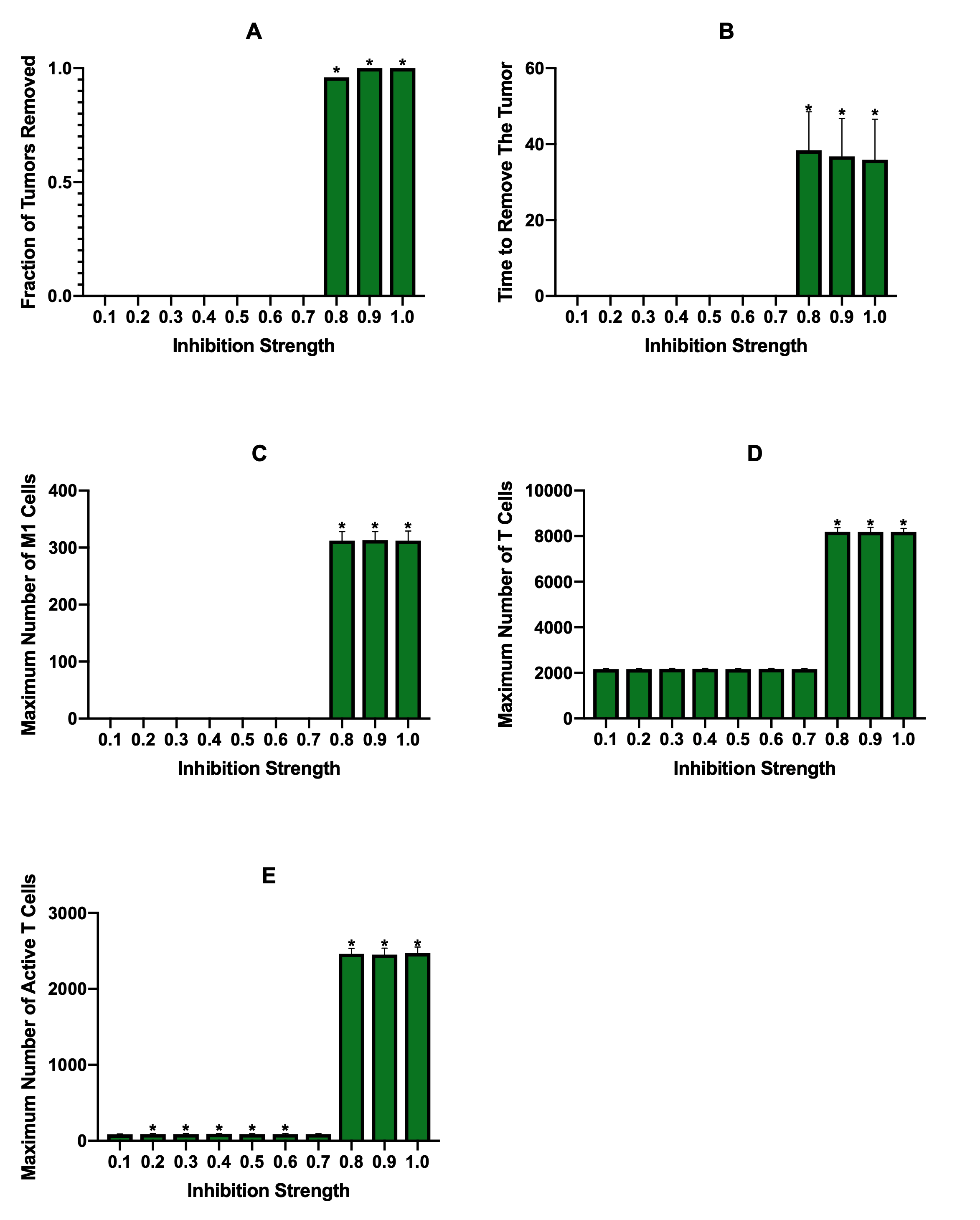

Supplement: S13 Fig — (A) fraction of tumors removed after starting simulation, (B) average time needed to remove the tumor, (C) the maximum number of M1 macrophages, (D) the maximum number of total T cells, (E) the maximum number of active T cells. Note the differences in y-axis scales across treatment strategies. Asterisks signify that a result is statistically significant (p<0.01) from the result of the lowest treatment strength. We note that for the time needed to remove the tumor (B), plotted is the time averaged over only simulations where the tumor was removed. Therefore, while some bars may appear much higher than that of the lowest treatment strength, they only represent a small number of simulations out of 100 and thus were not found to be statistically significant. (TIF) [file pcbi.1008519.s013.tif]

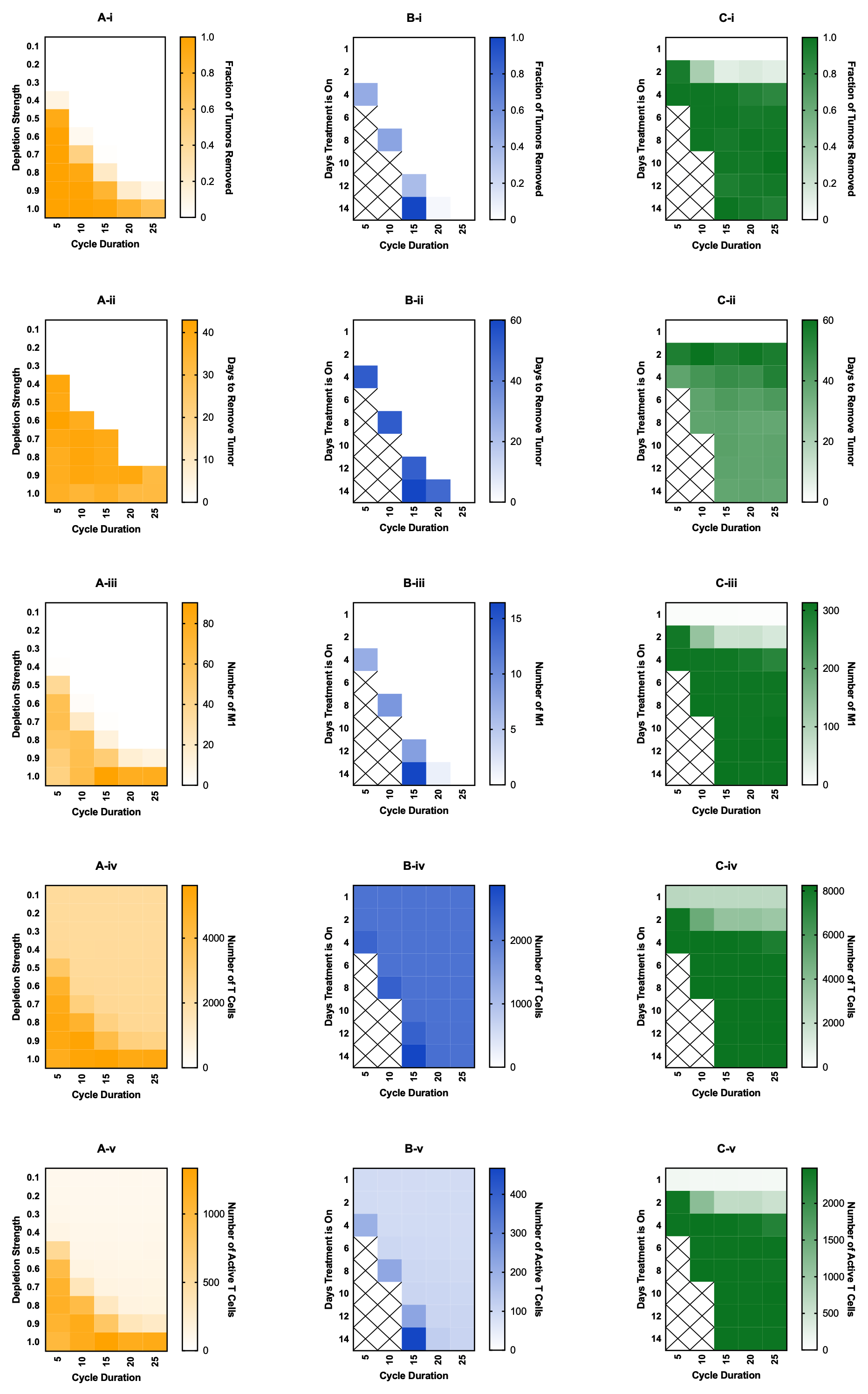

Supplement: S14 Fig — For macrophage depletion (A), the fraction of macrophages removed at the beginning of each cycle is given as “Depletion Strength” and the length of each cycle is “Cycle Duration.” For recruitment inhibition (B) and PI3K inhibition (C), the number of days in the cycle that treatment is on for is given as “Days Treatment is On.” Recruitment inhibition is simulated at a strength of 1.0 (complete inhibition) and PI3K inhibition is simulated at a strength of 0.8. For recruitment inhibition and PI3K inhibition, spaces marked with an X are those where treatment-on time is equal or greater to the cycle duration, thus were not simulated. (i) fraction of tumors removed after starting therapy. (ii) time (days) from starting treatment to tumor removal. It is averaged over the 100 simulations and is equal to zero if no tumors were removed at that treatment level. (iii) maximum number of M1 macrophages. (iv) maximum number of total T cells. (v) maximum number of active T cells. (TIF) [file pcbi.1008519.s014.tif]

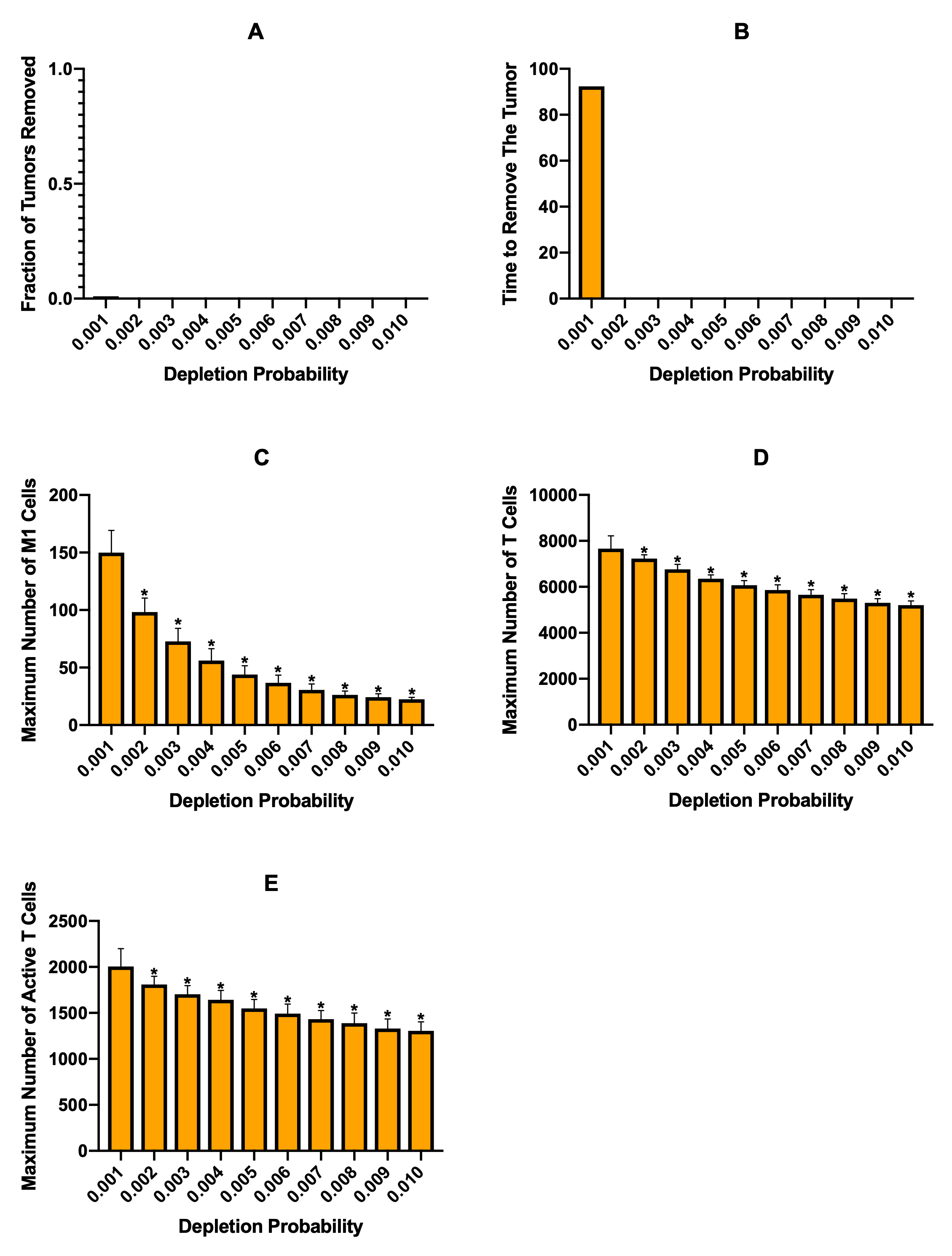

Supplement: S15 Fig — (A) fraction of tumors removed after starting simulation, (B) average time needed to remove the tumor, (C) the maximum number of M1 macrophages, (D) the maximum number of total T cells, (E) the maximum number of active T cells. Note the differences in y-axis scales across treatment strategies. Asterisks signify that a result is statistically significant (p<0.01) from the result of the lowest treatment strength. We note that for the time needed to remove the tumor (B), plotted is the time averaged over only simulations where the tumor was removed. Therefore, while some bars may appear much higher than that of the lowest treatment strength, they only represent a small number of simulations out of 100 and thus were not found to be statistically significant. (TIF) [file pcbi.1008519.s015.tif]

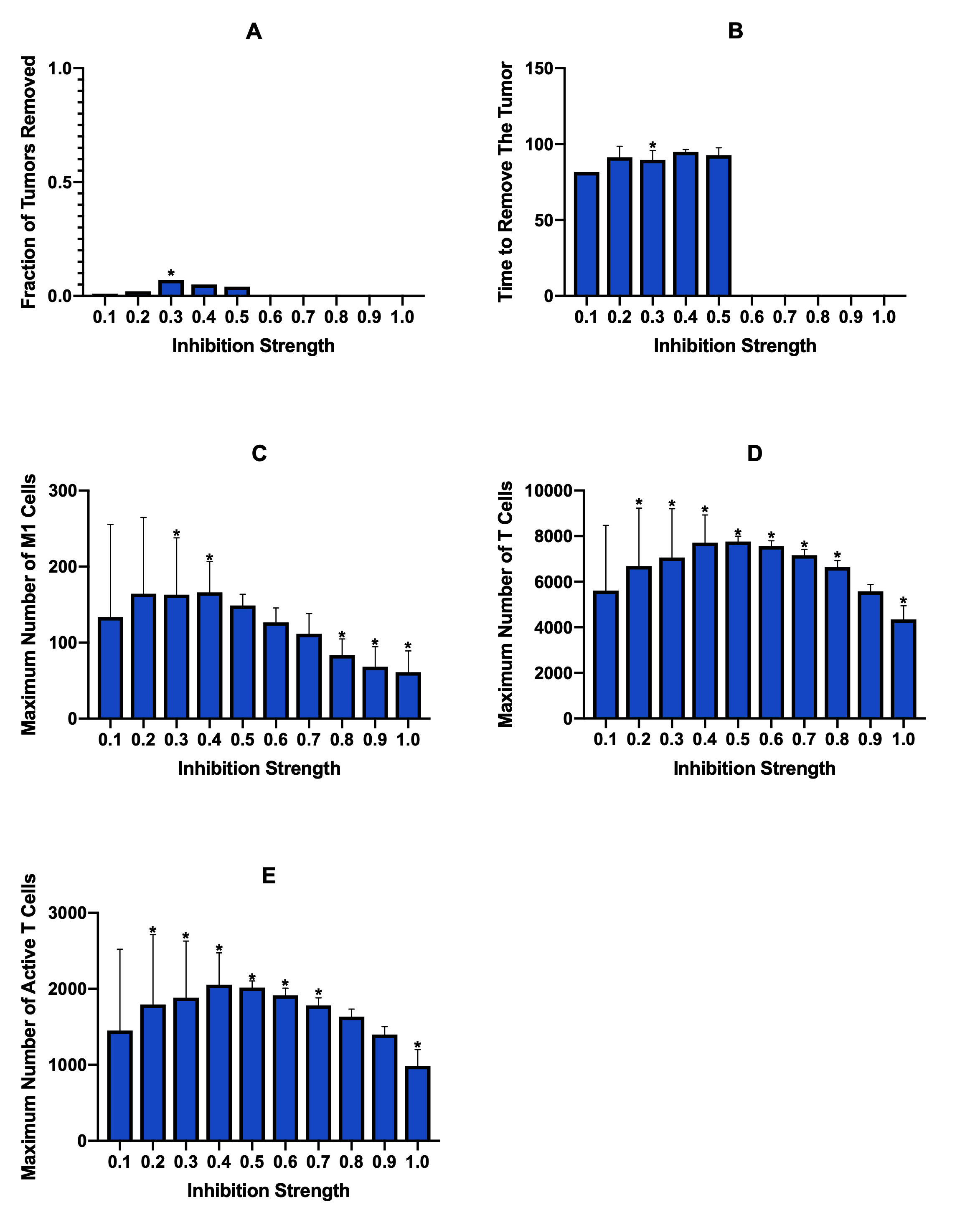

Supplement: S16 Fig — (A) fraction of tumors removed after starting simulation, (B) average time needed to remove the tumor, (C) the maximum number of M1 macrophages, (D) the maximum number of total T cells, (E) the maximum number of active T cells. Note the differences in y-axis scales across treatment strategies. Asterisks signify that a result is statistically significant (p<0.01) from the result of the lowest treatment strength. We note that for the time needed to remove the tumor (B), plotted is the time averaged over only simulations where the tumor was removed. Therefore, while some bars may appear much higher than that of the lowest treatment strength, they only represent a small number of simulations out of 100 and thus were not found to be statistically significant. (TIF) [file pcbi.1008519.s016.tif]

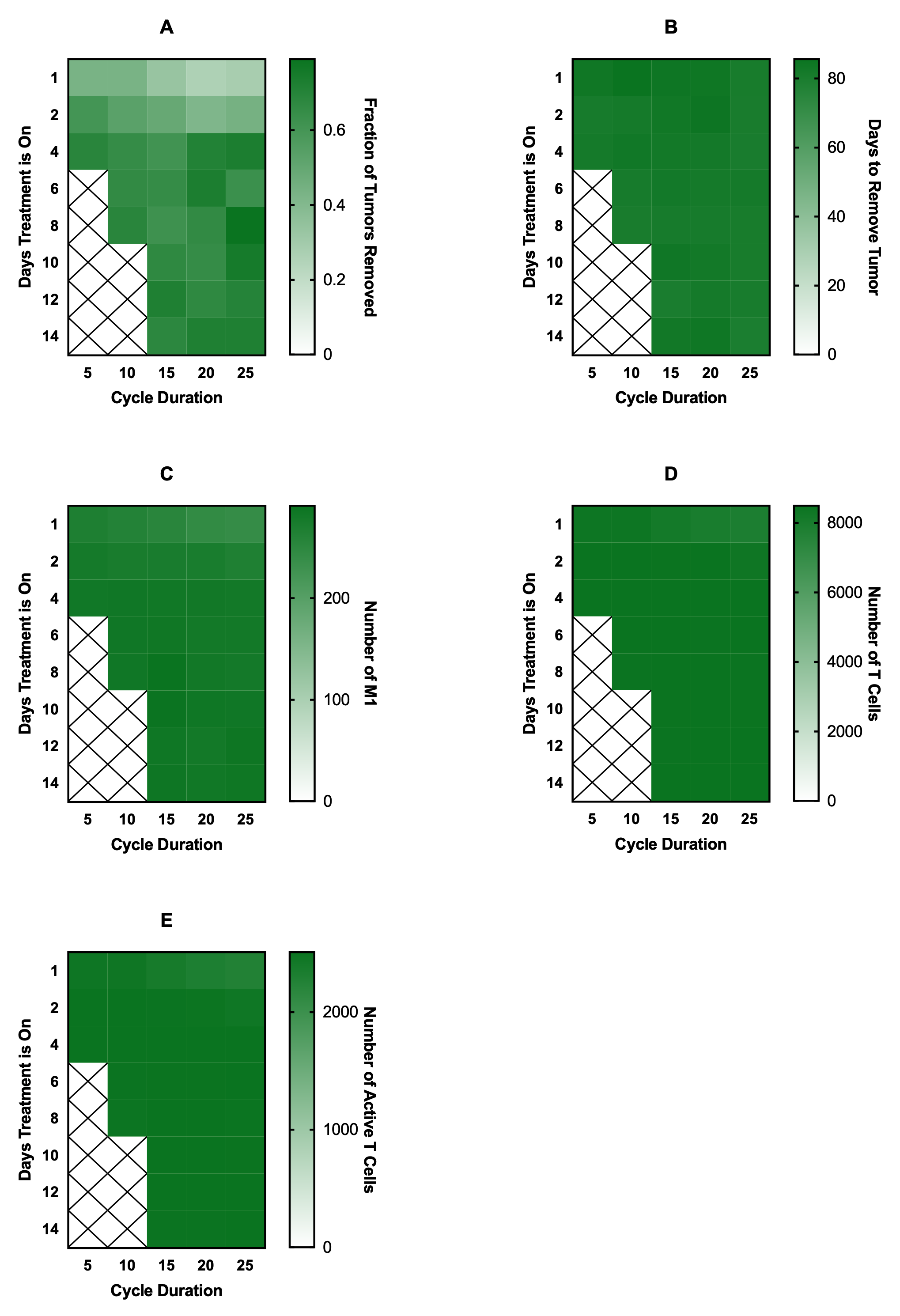

Supplement: S17 Fig — The macrophage recruitment rate is doubled, and tumor proliferation rate increased to 1.2/day. (A) fraction of tumors removed after starting simulation, (B) average time needed to remove the tumor, (C) the maximum number of M1 macrophages, (D) the maximum number of total T cells, (E) the maximum number of active T cells (TIF) [file pcbi.1008519.s017.tif]

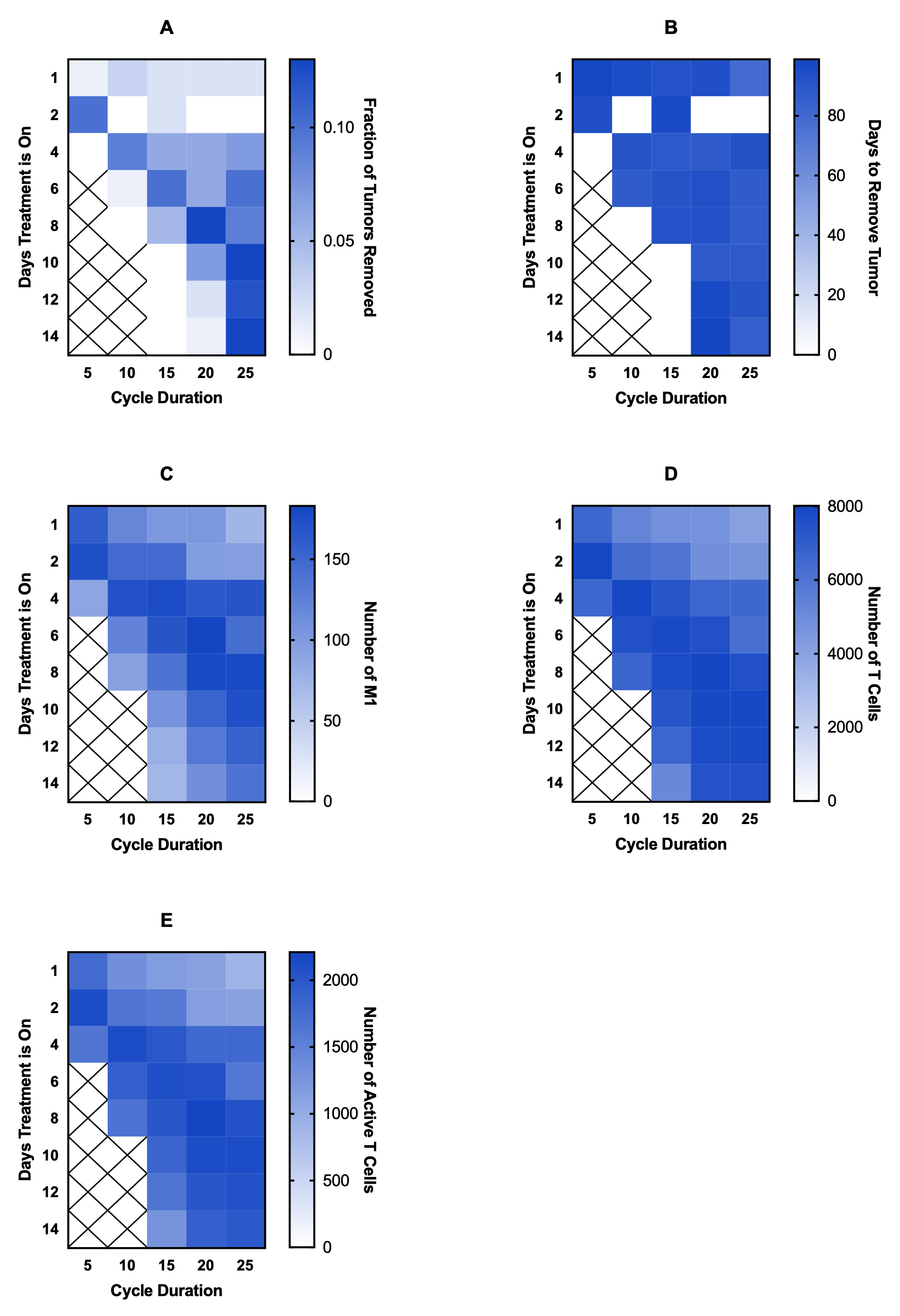

Supplement: S18 Fig — The macrophage recruitment rate is doubled, and tumor proliferation rate increased to 1.2/day. (A) fraction of tumors removed after starting simulation, (B) average time needed to remove the tumor, (C) the maximum number of M1 macrophages, (D) the maximum number of total T cells, (E) the maximum number of active T cells (TIF) [file pcbi.1008519.s018.tif]
