# Supplementary material for: Multi-scale modeling of macrophage—T cell interactions within the tumor microenvironment
Source: PLoS Comput Biol. 2020 Dec 23;16(12):e1008519. doi: 10.1371/journal.pcbi.1008519 (PMC7790427; doi:10.1371/journal.pcbi.1008519)
Supplement: S1 Table — Parameter values, with supporting references. For the tumor division time and macrophage recruitment rate parameters, values shown in parentheses are the increased values used for certain simulations, as described in the Results. (DOCX) [file pcbi.1008519.s019.docx]

**S1 Table. Model Parameters**

| **Category** | **Parameter** | **Value** | **Reference** |
| --- | --- | --- | --- |
| T Cell Recruitment | *tdelay* | 5 days | [1] |
|  | *twindow* | 1 day | [1] |
|  | *ka* | 15 (dimensionless) | [1] |
|  | *ki* | 0.01 (dimensionless) | [1] |
|  | *r1* | 6 cells/hr | [1] |
| Diffusion | Diffusion constant | 300 μm^2^/sec | [2] |
|  | lattice size | 15 μm | [3] |
| Cancer Cells | Tumor division time | 30 hrs (20 hrs) | [1], [4-10] |
|  | Tumor cell lifespan | 5 days | [1] |
|  | Tumor cell - T cell killing time | 6 hrs | [4] |
|  | Macrophage activating factor secretion | 1×10^-7^ pg/sec | Adapted from [6] |
| T cells | T cell division time | 8 hrs | [1] |
|  | T cell lifespan | 41 hrs | [5] |
|  | T cell maximum number of kills | 5 tumor cells | [4] |
| Macrophages | Macrophage lifespan | 30 days | [11,12] |
|  | Macrophage recruitment rate | 1×10^-8^ cells/(site x sec)  (2×10^-8^) | [6] |
|  | Initial number of macrophages | 2×10^-3^ cells/site | [6] |
|  | Macrophage activation threshold | 8×10^-6^ pg/site | [6] |
| Cytokine Secretion | Tumor cell IL-4 secretion | 10 mol/sec | [13] |
|  | M2 IL-4 secretion | 10 mol/sec | [13] |
|  | T cell IFNγ secretion | 26 mol/sec | [13] |
| T cell activation | *k* | 3 (dimensionless) | model specific |
|  | *s* | 0.5 (dimensionless) | model specific |
| Other | Time step | 0.5 hrs | model specific |
|  | Cell migration speed | 1 site/time step | [6] |

**References**

1. Gong C, Milberg O, Wang B, Vicini P, Narwal R, Roskos L, et al. A computational multiscale agent-based model for simulating spatio-temporal tumour immune response to PD1 and PDL1 inhibition. J R Soc Interface. 2017;14(134):20170320.
2. Wells DK, Chuang Y, Knapp LM, Brockmann D, Kath WL, Leonard JN. Spatial and functional heterogeneities shape collective behavior of tumor-immune networks. PLoS Comput Biol. 2015;11(4):e1004181.
3. Hao S-J, Wan Y, Xia Y-Q, Zou X, Zheng S-Y. Size-based separation methods of circulating tumor cells. Adv Drug Deliv Rev. 2018;125:3–20.
4. Kather JN, Poleszczuk J, Suarez-Carmona M, Krisam J, Charoentong P, Valous NA, et al. In silico modeling of immunotherapy and stroma-targeting therapies in human colorectal cancer. Cancer Res. 2017;77(22):6442–6452.
5. Kim PS, Lee PP. Modeling protective anti-tumor immunity via preventative cancer vaccines using a hybrid agent-based and delay differential equation approach. PLoS Comput Biol. 2012;8(10):e1002742.
6. Wells DK, Chuang Y, Knapp LM, Brockmann D, Kath WL, Leonard JN. Spatial and functional heterogeneities shape collective behavior of tumor-immune networks. PLoS Comput Biol. 2015;11(4):e1004181.
7. Mahlbacher G, Curtis LT, Lowengrub J, Frieboes HB. Mathematical modeling of tumor-associated macrophage interactions with the cancer microenvironment. J Immunother Cancer. 2018;6(1):1–17.
8. Knutsdottir H, Condeelis JS, Palsson E. 3-D individual cell based computational modeling of tumor cell–macrophage paracrine signaling mediated by EGF and CSF-1 gradients. Integr Biol. 2016;8(1):104–119.
9. Knútsdóttir H, Pálsson E, Edelstein-Keshet L. Mathematical model of macrophage-facilitated breast cancer cells invasion. J Theor Biol. 2014;357:184–199.
10. Norton K-A, Jin K, Popel AS. Modeling triple-negative breast cancer heterogeneity: Effects of stromal macrophages, fibroblasts and tumor vasculature. J Theor Biol. 2018;452:56–68.
11. Boissonnas A, Laviron M. Ontogeny of tumor-associated macrophages. Front Immunol. 2019;10:1799.
12. Parihar A, Eubank TD, Doseff AI. Monocytes and macrophages regulate immunity through dynamic networks of survival and cell death. J Innate Immun. 2010;2(3):204–215.
13. Han Q, Bradshaw EM, Nilsson B, Hafler DA, Love JC. Multidimensional analysis of the frequencies and rates of cytokine secretion from single cells by quantitative microengraving. Lab Chip. 2010;10(11):1391–1400.
