# Supplementary material for: Multi-scale modeling of macrophage—T cell interactions within the tumor microenvironment
Source: PLoS Comput Biol. 2020 Dec 23;16(12):e1008519. doi: 10.1371/journal.pcbi.1008519 (PMC7790427; doi:10.1371/journal.pcbi.1008519)
Supplement: Attachment — Submitted filename: Response to reviewers_R1.docx [file pcbi.1008519.s021.docx]

**Response to reviewers**

We thank the editor for the opportunity to provide a revised manuscript. We appreciate the reviewers’ constructive comments (blue text). We have worked to address their comments and submit an improved the manuscript. Below, we provide a detailed response to each comment and indicate how we have edited the manuscript text to address the comments (black text). Additionally, we provide a file that highlights the changes made to the manuscript text. The line numbers mentioned below correspond to the manuscript with changes tracked.

**Comments from Reviewer 1**

Fig 1: caption mentions green dots (M0 macrophages), but figure does not seem to have green dots. The figure, however, seems to show blue dots.

We have edited this figure to have a background white, which better shows the various cells: tumor cells (black), T cells (yellow), active T cells (red), M0 macrophages (blue), and M2 macrophages (purple).

Fig 2: caption mentions red for tumors removed, but there seems to be no red in the figure (or perhaps it is barely visible?)

There is a single red trace for the one tumor that was removed under this condition. We have made the line for that curve thicker to make it more visible and edited the caption to mention there is a single red trace (Lines 221-222).

Fig 3: caption mentions A, B, and C, as well as i, ii, iii, and iv, but none of these labels are in the figure. Further, most of the results do not seem to show statistically significant differences. Could the results perhaps be summarized more succinctly?

The columns correspond to A, B, and C, while the rows are numbered from i to v. The labeling with letters and Roman numerals is given in the heading above each panel, but perhaps the labels were not easily seen by the reader. We have made the headings more prominent by placing a dash between the letters and Roman numerals.

Fig 5: caption mentions tumors that survived to be in black color, yet none are shown in the figure. Further, many of the orange tumor sequences seem to be essentially the same as in Fig 4. Is there a need to show Fig 5 or can the results be stated in words?

For the value of the probability depletion used for this figure, all tumors removed. The caption should have been updated to reflect that. This figure has been moved to S5 Fig. We have left it in to explain the increase in M1 macrophages seen in Fig 3A.

Fig 6: caption mentions A, B, and C, as well as i, ii, iii, and iv, but none of these labels are in the figure. Further, the panels do not seem to match what the caption is saying (or if it is, it seems confusing). The units for the colors should be given.

The columns correspond to A, B, and C, while the rows are numbered from i to v. The labeling with letters and Roman numerals is given in the heading above each panel, but perhaps the labels were not easily seen by the reader. We have made the headings more prominent by placing a dash between the letters and Roman numerals. Additionally, we have provided units for the color bars. Note this is now Fig 4.

Fig 9: caption mentions A, B, and C, as well as i, ii, iii, and iv, but none of these labels are in the figure. Most panels seem to show non-significant differences.

The columns correspond to A and B, while the rows are numbered from i to v. The labeling with letters and Roman numerals is given in the heading above each panel, but perhaps the labels were not easily seen by the reader. We have made the headings more prominent by placing a dash between the letters and Roman numerals. Note this figure has been replaced by Fig 7 and Fig 11.

Fig 12: red lines are barely visible.

We have made the line for red curve thicker to make it more visible.

Fig 13: units for color scales should be given

We have edited the figure to provide units for the color bars.

Lastly, for those results that are statistically significant, comparisons with appropriate statistics would help to interpret the differences, especially given the stochastic nature of the studies.

We have edited the bar graphs to indicate cases that are statistically different from lowest treatment.

**Comments from Reviewer 2**

Reproducibility report has been uploaded as an attachment.

We have edited the model files to address the comments from the reproducibility report. The main edit is to make the files immediately executable without changes by the user.

**Comments from Reviewer 3**

1. The manuscript would benefit from additional discussion, speculation, or insight to help interpret some of the unintuitive results, such as why moderate treatment was preferred in cases where tumor proliferation and macrophage recruitment rates were both increased.

We have expanded the Discussion section to provide some insight to explain the modeling result that moderate treatment is more effective for these cases (lines 634-640).

2. Please be more explicit when supporting the claim that previously reported experimental results are comparable to the results found here. How do those experimental setups compare to the model used here? Did these experiments take place in a tissue with vasculature or in a dish? Were there nutrient limitations? Please describe exactly which trends and observations are used as model validation or verification in this study.

We qualitatively compared simulation results to specific *in vivo* experiments in both mouse and human studies. These comparisons have been added to Discussion section at lines 645-659.

3. The use of the neural net to avoid explicitly model intracellular signaling is innovative and interesting. However, no data nor analysis is presented to validate this model simplification; this gap should be addressed.

We thank the reviewer for pointing out this missing information. After splitting the simulated dataset from the ODE model into training and testing sets, we found that the neural network achieves >98% prediction accuracy on the testing set. From this, we concluded that for our purposes of classifying cell response into discrete categories, the neural network is an adequate model simplification. We have increased the detail in the Methods section at lines 797-803.

4. One limitation of the model seems to be the lack of accounting for nutrients or nutrient limitations, which cause common features of tumors such as hypoxia and necrotic cores. While the authors do not necessarily need to add this phenomenon to their model, this should be discussed as a limitation, and artifacts arising from this choice should be considered.

We agree with the reviewer that including additional model features, such as nutrient availability and consumption, can enhance the model. For the current study, we made the decision not to include nutrients to the model due to the additional complexity involved. While hypoxia does impact both macrophages and T cells, we wanted to focus on the interactions between the two immune cell types, and adding in another layer of complexity would have prompted us to perform several other sets of simulations that we felt would be better left for a separate in-depth study. We have included this limitation in the Discussion at lines 687-695.

5. Related to the last point, it would be worth investigating whether the factors that limit tumor growth are truly immunological control vs. nutrient limitation, space limitation, etc. It does not seem obvious that a rather arbitrarily parameterized model would yield a scenario in which immune control is achieved.

This is a good point, and we thank the reviewer for raising it. To address this comment, we simulated the tumor with immune cells present, but without immune function, and compared the tumor growth curves to tumor growth curves without any immune cells present. We found that the growth is essentially the same, showing that immune control is the reason for reaching a steady state and not spatial limitations. This has been added to the Results at line 205.

6. If each lattice site fits one agent, this is concerning because cancer cells have a far larger volume than do T-cells, and it is possible that this could impact both replication and motility. The authors must provide argument or evidence that this isn’t a problem.

This is a good point that we have addressed in the revised submission. We can point to many other models that assume one cell per site, even with cells of different sizes. We have addressed this limitation in the Methods at lines 717-720.

7. It would be interesting to see an analysis of the spatial distribution of the cell types throughout the simulation. Treating solid tumors is complicated because it can be difficult for immune cells to penetrate the tumor core. An analysis like this would be useful in showing that the model has the capability to collect data that is difficult to collect in vitro or in vivo but that is relevant, important, and interesting.

We appreciate the reviewer’s point; however, we believe that an extensive quantitative analysis of the spatial distribution of the cells would be fairly complex and warrants its own study. For the present manuscript, we have added a description of the spatial distribution of the cells to the Results at lines 160-164 and mentioned this as an avenue for future research at lines 693-695.

8. Please clarify how treatment cycling, as modeled here, relates to clinical scenarios of relevance to patient treatment practices. Is this simply a matter of patient compliance or is there more to this phenomenon?

There are multiple factors that could prevent the continuous administration of a treatment, including patient compliance, as the reviewer mentions. In this work, we are mainly concerned with the potential for adverse effects, which would be a reason for lower levels of treatment. We have expanded upon this in the Results at lines 376-378.

9. The authors mention that for constant vs cycled treatment, they change the probability of macrophage recruitment in order to avoid complete depletion of macrophages in the constant treatment case. This raises concerns about making a fair comparison between continuous vs cycled treatment, as this probability change introduces bias. Please justify why this comparison is fair; the given statement “If it were as the same strength in both cases, the constant treatment would basically get treated immediately” is insufficiently precise.

For macrophage depletion at a continuous rate, it is necessary to only consider a small probability of apoptosis because that probability of death occurs at every time step, which would lead to complete and rapid removal of the macrophages over the course of the simulation. To set the range of depletion probabilities, we decreased the probability until we reached a value that had little tumor removal, so that we could compare an ineffective level of treatment to more effective levels. This change also allows us to be able to compare results to the cycled treatment, where macrophages are removed more slowly, and to isolate the effects of cycled versus continuous treatment. We provide this explanation in the Methods at lines 904-907.

10. The authors report “data not shown” for a few data sets, concerning treatment of specifically altered types of tumors; these data are interesting and should be included in the supplement.

We have included these additional results in the supplement (S9 Fig. – S18 Fig.).

11. In one part of this study, there seems to be a gap in the analysis presented. It would be helpful to evaluate PI3K treatment efficacy in the case where only macrophage recruitment rate in the tumor was changed, since this treatment was evaluated for tumors with only tumor proliferation rate altered or with both tumor proliferation rate and macrophage recruitment rate changed. This, the suggested evaluation seems like a missing case/control.

We have included these results in the manuscript (S13 Fig and Fig 12).

12. Showing the PI3K inhibition data for tumors with both tumor proliferate rate and macrophage recruitment rate altered (Fig 9B) far before discussing these data is confusing; it might be more clear if these data were moved to later to match the text.

In the revised manuscript, we have moved Figure 11 to improve the flow.

13. The figures and data presentation could be substantially improved by considering a few general suggestions:

a. Include labels within figures to annotate panels/columns and scale bars (i.e., tumor removal, cancer cell count, etc.) to indicate what is being measured without requiring the reader to consult the figure caption

We have edited the figures to have the panel labels be more prominent to aid the reader.

b. Normalizing scale bars and graph axes that measure the same thing across treatment types would make it easier to compare outcomes across treatments

We thank the reviewer for this suggestion. Since the scales are quite different across the figure panels showing the model predictions, making the y-axes show the same scales would make some results unreadable. Therefore, we have edited the captions to note the different axes limits.

14. In general, presenting simulation results as a series of trajectories provides a nice way to view the distribution of outcomes within any one panel. However, it does not enable one to evaluate the correlations/relationships between the data presented in any two (or more) panels. Would it be possible to include more rigorous calculations to evaluate such relationships (e.g., to back up or test statements such as “outcome X tends to occur when behavior Y is observed”, or to evaluate positive and negative correlations described in the text)?

We agree that a more in-depth analysis of the trajectories could provide useful insights. However, we feel that this extension is a bit beyond the scope of this study and would deserve its own study. We will look into pursuing this in the future.

15. Have you considered running a simulation without immune cells to evaluate the baseline dynamics of tumor size and immune-independent phenomena? This base case could enable comparison with some experimental systems that match this scenario.

We added this to the Results at line 205 and S3 Fig. It shows typical exponential growth.

Specific comments:

Line 56: Please clarify (up front) that macrophage functional polarization, while useful at a conceptual level, actually represents a continuum of phenotypes rather than discrete M1 vs M2 categorical distinctions. This is discussed at the end the paper but should be noted here, too.

We have added this point to the Introduction at lines 56-59.

Line 72-73: The authors note one cause of T-cell exhaustion. However, for completeness it may be useful to mention that T-cell exhaustion is also caused by excessive and continuous stimulation, not just checkpoint proteins.

Wherry, E. J., & Kurachi, M. (2015). Molecular and cellular insights into T cell exhaustion. Nature Reviews Immunology, 15(8), 486–499. <https://doi.org/10.1038/nri3862>

We have added this point and corresponding reference to the Introduction at lines 75-78.

It is possible that the phenomenon of exhaustion is captured by M2 macrophages preventing neighboring T-cells from becoming active. What happens to T-cells after they reach their 5 kill max limit? Is that supposed to be T-cell exhaustion? The authors should be clearer about how/whether their model describes T-cell exhaustion.

At the maximum kill limit, T cells because exhausted. This means they are still alive, but no longer function. We have clarified this point in the Methods at lines 858-859.

Line 80: It could be worth noting that more recent findings suggest that blocking inhibitory signals does not actually recover exhausted T-cells but it might help new T-cells infiltrate the tumor and prevent them from becoming exhausted.

Yost, K. E., Satpathy, A. T., Wells, D. K., Qi, Y., Wang, C., Kageyama, R., … Chang, H. Y. (2019). Clonal replacement of tumor-specific T cells following PD-1 blockade. Nature Medicine, 25(8), 1251–1259. <https://doi.org/10.1038/s41591-019-0522-3>

We have added this point and the corresponding reference to the Introduction at lines 87-90.

Line 116: Consider noting and discussing the following ABM paper, which includes many cell types interacting at a site of infection (not necessarily a tumor, but might be worth noting)

Folcik, V. a, An, G. C., & Orosz, C. G. (2007). The Basic Immune Simulator: an agent-based model to study the interactions between innate and adaptive immunity. Theoretical Biology & Medical Modelling, 4, 39. https://doi.org/10.1186/1742-4682-4-39

We have added this point and the corresponding reference to the manuscript at lines 127-128.

Line 165: Figure S1: Please elaborate upon the statement that the parameters besides macrophage recruitment rate seem to have sharp peaks in seemingly random places; why might this be the case?

We used Latin Hypercube Sampling (LHS) to explore a range of parameter values. LHS generates a series of parameter sets where each parameter value only appears once. Therefore, in this figure, we see sharp spikes due to each parameter value being simulated once, and not the average response at the parameter value. Despite this, we still see a clear trend in regard to macrophage recruitment rate. We have edited the text to better explain this point (lines 180-184).

Line 170: “TAMS” should be “TAMs”

We have made this change (line 191).

Line 182: Please briefly explain why T cells are introduced at a specific timepoint (this is also not clear from the model description).

The equation for T cell recruitment includes a delay parameter to account for T cell trafficking. We added this description to the Methods at lines 820-824.

Line 182: 350 cells seems like a very small tumor. What is the total area/volume this tumor covers? 4000 molecules of IL-4 also seems somewhat small? Given the number of macrophages, it seems like this would be higher? The authors should consider justifying the number of each cell types or at least relative fractions of each that the model finds as an outcome as part of model validation.

The model represents a small initial metastasis. It is also in 2D, representing a tissue slice instead of the entire tumor. Thus, it is difficult to compare relative cell fractions because this model is for early growth, whereas any published data on cell fractions is for a large tumor that has had a long time (even up to several years) to influence its local environment. We have provided more explanation to address the reviewer’s point at lines 712-713.

Line 202: “effects” should be “affects” since it’s the verb

We have made this change (line 230).

Line 213: To clarify, does this treatment remove macrophages of each kind (M0, M1,M2) with equal probability? This was later discussed but it would be useful to clarify here where it is defined.

All macrophage subtypes are eliminated with equal probability. We thank the reviewer for raising this point, which has been clarified at line 243.

Line 214: “s-axis” should probably be “x-axis”

We have made this change (line 242).

Line 213-225: These data require a bit more investigation. It is not clear how increasing the depletion probability from 0.001 to 0.002 yields a dramatic increase in tumors removed while conferring no discernible impact on the max number of M1 cells or the max number of activated T cells. The authors appropriately avoid drawing a strong conclusion, but even the general mechanism proposed does not explain this initial sharp rise. Is it possible that this phenomenon arises due to competition for space on the lattice?

We have elaborated on the explanation as to the sharp change in the fraction of tumors removed for these two cases in lines 247-250. We also investigated the effect of spatial competitions, as noted in response to point 5 from Reviewer 3: “we simulated the tumor with immune cells present, but without immune function, and compared the tumor growth curves to tumor growth curves without any immune cells present. We found that the growth is essentially the same, showing that immune control is the reason for reaching a steady state and not spatial limitations. This has been added to the Results at line 205.”

Line 219: Can this be called biphasic, given the error? The term biphasic is a bit too strong of a claim and doesn’t add to your argument.

We appreciate the reviewer’s point and have changed the wording to refer to it as a peak. This point is clarified at lines 251-260.

Line 282: Could you do a spatial analysis to try to prove or disprove this claim? For example, is it possible (or useful) to compare spatial distributions of each cell type between cases where the tumor is eliminated vs. not?

As given in response to point 7 above, we appreciate the importance of the cells’ spatial distribution. However, an extensive quantitative analysis of the spatial distribution of the cells would be fairly complex and warrants its own study. For the present manuscript, we have added a description of the spatial distribution of the cells to the Results at lines 160-164 and mentioned this as an avenue for future research at lines 693-695.

Line 284: Why do you think that no M1s appear here in this treatment but do appear in the depletion treatment if both treatments have the overall effect of lowering the number of macrophages in the simulation? Please clarify this reasoning.

Macrophages migrate towards the tumor, where there are higher levels of IL-4 keeping them in the M2 state. Depletion reduces the number of macrophages by removing current macrophages, with new macrophages continuing to enter the simulation far enough away from the tumor where they can polarize to M1. Recruitment inhibition lowers the number of macrophages by preventing new macrophages from entering the system, so that current macrophages remain close to the tumor and retain the M2 state. We have added this description to the Results at lines 331-337. We thank the reviewer for raising this point.

Line 291: The claim that PI3K inhibition showed the fastest removal time seems to be too strong as that only seems to be the case for one of the bars shown; otherwise, column B seems to have faster removal times on average for cases where the treatment was effective. However, this comparison is difficult to make visually because the axes all have different ranges. Please scale the axes to have the same range and max (across rows) for easier visual comparison (or at least in all the ii graphs).

We have adjusted the figure captions to address this point. It is difficult to have the same axis ranges because of the vastly different results of the treatment strategies. However, we have noted the different scales in the figure captions to draw the reader’s attention to this point.

Line 418: Where is the data for cycling PI3K inhibition? Please add a figure citation.

We have included these results in S9 Fig.

Line 422: Where is the data for PI3K inhibition for tumors with macrophage recruitment rate doubled and unchanged tumor proliferation rate? Please add a figure citation.

We have included these results in S13 Fig.

Line 445: The claim that this response in 11C is biphasic does not seem justified, as it seems too strong. It could easily be a left-shifted single peak.

We appreciate the reviewer’s point and have changed the wording to refer to it as a “peak”. This point is clarified at lines 523-524.

Line 456: In the sentence “behave similarly to that case”, to what does “that” refer? This behavior appears to be a mix of the behaviors seen in the increased tumor proliferation only case and the increased macrophage recruitment rate only case.

The behavior is similar to the previous simulations without treatment. We have clarified this point at line 541.

Line 473: These data would be useful to show in the supplement, since a key goal of this paper is investigating how these treatments work across tumor types (i.e., tumors with varying parameters).

We have included these results in S15-16 Fig.

Like 497: It would be nice to show this result (treatment efficacy vs. equilibrium state) on a graph for each treatment type.

We appreciate the reviewer’s point; however, we believe that the graph would become crowded due to having to account for different treatment strengths and cycles, diminishing the reader’s understanding.

Line 617: How many macrophages are initialized in the tumor at the start of a simulation? This is not clearly defined in the methods and should be for the purpose of reproducibility and clarity. Additionally, if the authors have not done so already, please consider evaluating how changing this number impacts treatment outcomes.

The initial macrophage density is 2×10^-3^ cells/site (Table S18), and with a 100x100 grid, the starting number of macrophages is 20. We explored the impacts of changing the initial macrophage density and found that while it slightly changed the initial tumor dynamics, it had no bearing on long-term dynamics. This has been added to the Results at lines 216-218 and a new supplementary figure is added (S4 Fig).

Fig. 1B: It is very hard to see the green color with the gray background. Please also consider recoloring to accommodate readers who are red/green color blind.

We have edited this figure to make the background white, tumor cells (black), T cells (yellow), active T cells (red), M0 macrophages (blue), and M2 macrophages (purple).

Fig. 3: It seems that “Inhibition Strength” on the x-axes in Fig 3C’s are different than the “Inhibition Strength” on Fig 3B’s, but the authors might want to indicate that in some way (ex: “PI3K Inhibition Strength”); it’s currently confusing. This could be further clarified by the general suggestion to add labeling within figure/columns.

We have added more descriptive labels for the figure panels. We thank the reviewer for raising this point.

Fig. 6: Please label the scale bars on the figure. It would be useful to scale the color bars which depict the same quantity to be consistent across any one row.

We have edited the figure to a single range for the color bar to aid the reader in comparing results across simulated treatment modalities.

Fig. 7: Why are there distinct groups of tumors that respond in qualitatively similar ways but at different times? The authors should attempt an explanation of this observation, and it would be interesting even if it were simply speculation.

We have proposed an explanation for this model prediction, given at lines 429-432. Also, this figure is now Fig 5.

**Comments for Reviewer 4**

1. The title is a bit long and ambiguous...”and impacts of macrophage-based immunotherapies [on what?]”

We have shortened the title to be “Multi-scale modeling of macrophage – T cell interactions within the tumor microenvironment”, which encompasses the various simulations we performed, with and without macrophage-based treatment, for continuous and intermittent treatment cycles.

2. Line 214 refers to “s-axis”, presumably this is a typo and the authors mean “x-axis”.

We have made this changed at line 258.

3. Line 220 the authors state that the biphasic response in the maximum number of M1 macrophages is due to a decrease in IL-4 as tumor cells and M2 macrophages are removed, and it would be helpful to show plots of IL-4 to substantiate this claim.

In the revised manuscript, we now provide the time course of IL-4 to support the explanation as to the change in the maximum level of M1. This is present in S5 Fig.

4. Line 282 refers to spatial stochasticities but it is not clear to what extent the spatial nature of the model impacts the outcomes/predictions.

We have clarified this point at line 414. Here, we mean that, due to inherent randomness in the model, sometimes enough T cells are able to penetrate into the tumor to remove it and other times this does not happen. We have mentioned this in lines 345-346.

5. The focus of the paper is on predicting and evaluating the impact of adjusting macrophage dynamics, but a helpful “control” (counter-point) simulation would be to adjust T-cell numbers/dynamics.

We appreciate the reviewer’s comment and agree that T cell numbers and anti-tumor effects are important to study. We believe that expanding the paper to study T cell dynamics would increase an already lengthy paper. Additionally, by focusing on macrophage-based therapies, our paper complements a large body of modeling work that focuses on T cell dynamics. We have mentioned this point in the Discussion at lines 691-693.

6. Line 393 refers to “equilibrium state”, but it is not clear how the authors define this state. Line 496 refers to “equilibrium phase” – is this the same thing as “equilibrium state”?

We were using both “equilibrium state” and “equilibrium phase” to refer to the steady-state observed in the model, which is described in line 202. For clarity, we changed everything to “equilibrium state.” We thank the reviewer for pointing out this inconsistency.

7. The authors offer no attempt at model validation with independent experimental data and if it were possible to benchmark at least some of the predictions to experiments that would strengthen the credibility of the model.

We acknowledge that in the original manuscript submission, we did not include any comparison with experimental data or observations. It is very difficult to extensively compare our model to experimental data due to the inability to capture this data *in vivo*. Specifically, the model captures dynamics of very early tumor growth to understand how the tumor-immune ecosystem develops, but this is a scale largely inaccessible to quantitative experimental studies. However, we have added to the Discussion at lines 645-659 qualitative comparisons to *in vivo* human and mouse studies, which support our findings.
